# Supplementary figures and images for: Association between meat intake and mortality due to all-cause and major causes of death in a Japanese population
Source: PLoS One. 2020 Dec 15;15(12):e0244007. doi: 10.1371/journal.pone.0244007 (PMC7737902; doi:10.1371/journal.pone.0244007)

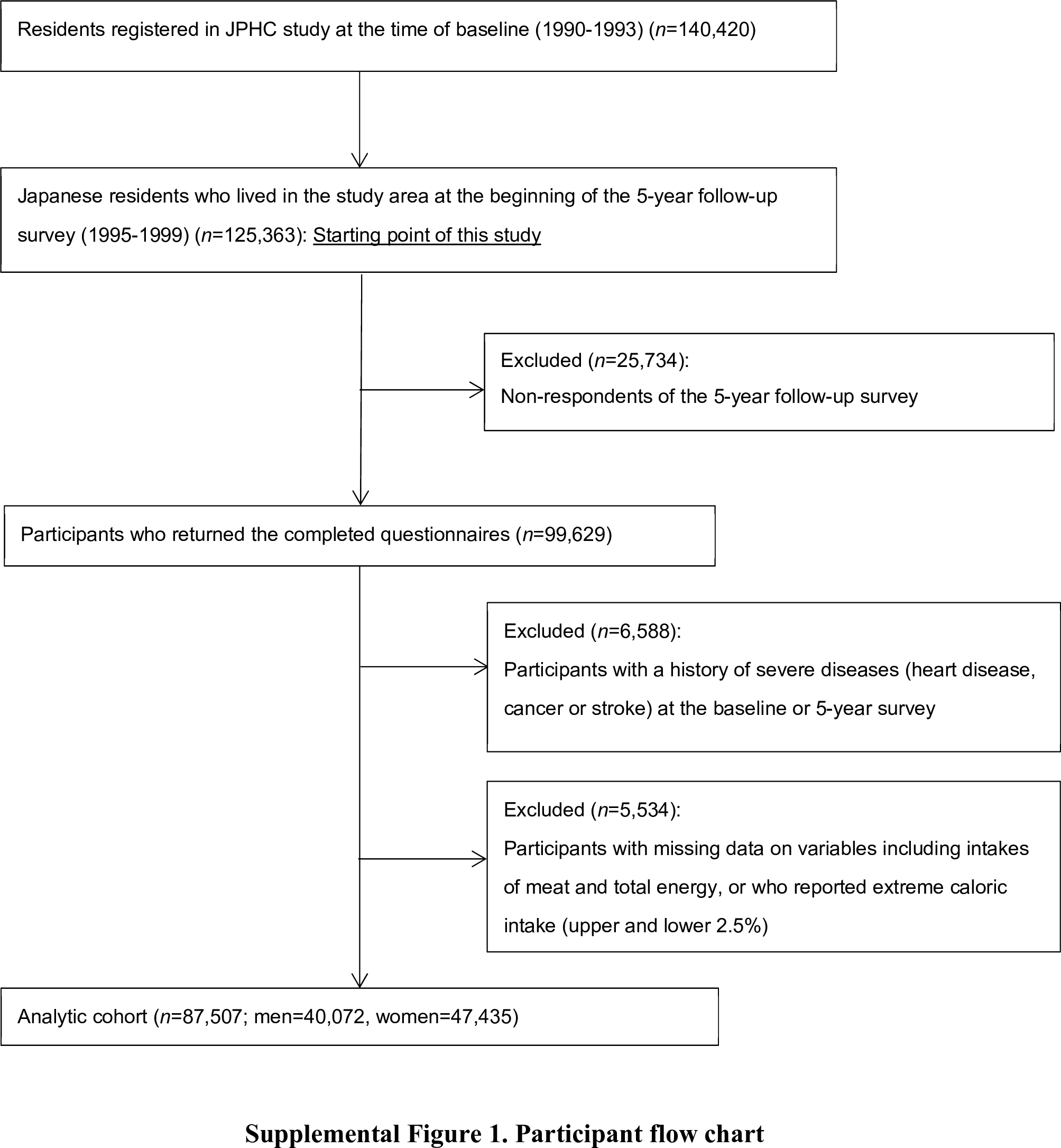

Supplement: S1 Fig — (TIF) [file pone.0244007.s001.tif]
