# Supplementary material for: Association between meat intake and mortality due to all-cause and major causes of death in a Japanese population
Source: PLoS One. 2020 Dec 15;15(12):e0244007. doi: 10.1371/journal.pone.0244007 (PMC7737902; doi:10.1371/journal.pone.0244007)
Supplement: S1 Table — (DOCX) [file pone.0244007.s002.docx]

**S1 Table. Adjusted hazard ratios of mortality by meat consumption status after excluding deaths within 5 years (men)**

|  | All-cause | | |  | Cancer | | | Cerebrovascular Disease | | |  | Heart Disease | | |  | Colorectal Cancer | | |
| --- | --- | --- | --- | --- | --- | --- | --- | --- | --- | --- | --- | --- | --- | --- | --- | --- | --- | --- |
|  | Cases | HR^1,2^ | 95% CI |  | Cases | HR^1,2^ | 95% CI | Cases | HR^1,2^ | 95% CI |  | Cases | HR^1,2^ | 95% CI |  | Cases | HR^1,2^ | 95% CI |
| All meat |  |  |  |  |  |  |  |  |  |  |  |  |  |  |  |  |  |  |
| Q1 | 1,415 | 1.00 |  |  | 549 | 1.00 |  | 147 | 1.00 |  |  | 174 | 1.00 |  |  | 62 | 1.00 |  |
| Q2 | 1,176 | 0.97 | (0.89-1.06) |  | 490 | 1.02 | (0.89-1.16) | 93 | 0.84 | (0.63-1.11) |  | 138 | 1.01 | (0.79-1.28) |  | 36 | 0.73 | (0.47-1.14) |
| Q3 | 1,166 | 1.03 | (0.94-1.13) |  | 492 | 1.10 | (0.95-1.26) | 110 | 1.07 | (0.79-1.44) |  | 124 | 1.01 | (0.77-1.33) |  | 32 | 0.68 | (0.41-1.11) |
| Q4 | 1,246 | **1.20** | **(1.07-1.35)** |  | 480 | 1.18 | (0.97-1.42) | 104 | 1.28 | (0.86-1.93) |  | 150 | **1.63** | **(1.15-2.29)** |  | 68 | 1.60 | (0.91-2.82) |
| *p for trend* |  | **0.008** |  |  |  | 0.077 |  |  | 0.232 |  |  |  | **0.037** |  |  |  | 0.345 |  |
| Red meat^3^ |  |  |  |  |  |  |  |  |  |  |  |  |  |  |  |  |  |  |
| Q1 | 1,424 | 1.00 |  |  | 551 | 1.00 |  | 144 | 1.00 |  |  | 174 | 1.00 |  |  | 57 | 1.00 |  |
| Q2 | 1,178 | 0.95 | (0.88-1.04) |  | 487 | 0.98 | (0.86-1.12) | 98 | 0.86 | (0.65-1.15) |  | 140 | 1.05 | (0.82-1.34) |  | 40 | 0.87 | (0.56-1.35) |
| Q3 | 1,170 | 1.03 | (0.94-1.12) |  | 499 | 1.11 | (0.96-1.28) | 107 | 1.14 | (0.83-1.55) |  | 113 | 1.00 | (0.76-1.33) |  | 36 | 0.85 | (0.52-1.39) |
| Q4 | 1,231 | **1.17** | **(1.03-1.32)** |  | 474 | 1.16 | (0.96-1.40) | 105 | 1.41 | (0.93-2.13) |  | 159 | **1.85** | **(1.31-2.62)** |  | 65 | 1.62 | (0.91-2.89) |
| *p for trend* |  | **0.022** |  |  |  | 0.072 |  |  | 0.095 |  |  |  | **0.009** |  |  |  | 0.234 |  |
| Beef^4^ |  |  |  |  |  |  |  |  |  |  |  |  |  |  |  |  |  |  |
| Q1 | 1,473 | 1.00 |  |  | 550 | 1.00 |  | 132 | 1.00 |  |  | 200 | 1.00 |  |  | 60 | 1.00 |  |
| Q2 | 1,205 | 1.01 | (0.93-1.09) |  | 495 | 1.06 | (0.93-1.20) | 118 | 1.22 | (0.94-1.60) |  | 140 | 0.89 | (0.71-1.12) |  | 46 | 0.97 | (0.64-1.47) |
| Q3 | 1,135 | 1.00 | (0.92-1.09) |  | 468 | 1.06 | (0.92-1.21) | 111 | 1.30 | (0.98-1.72) |  | 118 | 0.82 | (0.64-1.05) |  | 38 | 0.78 | (0.50-1.23) |
| Q4 | 1,190 | 1.06 | (0.97-1.16) |  | 498 | **1.20** | **(1.04-1.39)** | 93 | 1.14 | (0.82-1.57) |  | 128 | 0.95 | (0.73-1.24) |  | 54 | 1.14 | (0.73-1.79) |
| *p for trend* |  | 0.307 |  |  |  | **0.027** |  |  | 0.270 |  |  |  | 0.457 |  |  |  | 0.825 |  |
| Pork^5^ |  |  |  |  |  |  |  |  |  |  |  |  |  |  |  |  |  |  |
| Q1 | 1,385 | 1.00 |  |  | 526 | 1.00 |  | 152 | 1.00 |  |  | 173 | 1.00 |  |  | 52 | 1.00 |  |
| Q2 | 1,125 | 0.96 | (0.88-1.04) |  | 490 | 1.08 | (0.95-1.23) | 96 | 0.78 | (0.59-1.03) |  | 113 | 0.81 | (0.63-1.05) |  | 38 | 0.95 | (0.60-1.49) |
| Q3 | 1,195 | 1.01 | (0.92-1.11) |  | 489 | 1.08 | (0.94-1.25) | 99 | 0.83 | (0.62-1.12) |  | 145 | 1.09 | (0.84-1.40) |  | 43 | 1.08 | (0.68-1.72) |
| Q4 | 1,298 | **1.13** | **(1.02-1.26)** |  | 506 | **1.22** | **(1.03-1.44)** | 107 | 1.01 | (0.71-1.44) |  | 155 | 1.27 | (0.94-1.73) |  | 65 | 1.61 | (0.95-2.71) |
| *p for trend* |  | **0.028** |  |  |  | **0.037** |  |  | 0.893 |  |  |  | 0.075 |  |  |  | 0.087 |  |
|  |  |  |  |  |  |  |  |  |  |  |  |  |  |  |  |  |  |  |
| Processed meat^6^ |  |  |  |  |  |  |  |  |  |  |  |  |  |  |  |  |  |  |
| Q1 | 1,598 | 1.00 |  |  | 632 | 1.00 |  | 169 | 1.00 |  |  | 192 | 1.00 |  |  | 66 | 1.00 |  |
| Q2 | 1,174 | **0.91** | **(0.84-0.98)** |  | 461 | **0.84** | **(0.74-0.96)** | 108 | 0.82 | (0.63-1.06) |  | 144 | 1.04 | (0.82-1.30) |  | 43 | 0.88 | (0.58-1.34) |
| Q3 | 1,128 | 0.93 | (0.85-1.01) |  | 452 | 0.88 | (0.77-1.01) | 96 | 0.80 | (0.60-1.05) |  | 135 | 1.03 | (0.80-1.31) |  | 35 | 0.69 | (0.44-1.09) |
| Q4 | 1,103 | 0.97 | (0.89-1.06) |  | 466 | 0.99 | (0.85-1.14) | 81 | 0.81 | (0.59-1.11) |  | 115 | 0.99 | (0.75-1.30) |  | 54 | 1.02 | (0.65-1.60) |
| *p for trend* |  | 0.456 |  |  |  | 0.740 |  |  | 0.135 |  |  |  | 0.981 |  |  |  | 0.786 |  |
| Chicken^7^ |  |  |  |  |  |  |  |  |  |  |  |  |  |  |  |  |  |  |
| Q1 | 1,417 | 1.00 |  |  | 561 | 1.00 |  | 136 | 1.00 |  |  | 188 | 1.00 |  |  | 62 | 1.00 |  |
| Q2 | 1,228 | 0.97 | (0.90-1.05) |  | 514 | 0.99 | (0.88-1.12) | 106 | 0.87 | (0.66-1.14) |  | 138 | 0.86 | (0.68-1.08) |  | 42 | 0.79 | (0.52-1.20) |
| Q3 | 1,183 | 0.96 | (0.88-1.04) |  | 505 | 1.00 | (0.88-1.14) | 111 | 0.96 | (0.73-1.27) |  | 129 | 0.81 | (0.63-1.03) |  | 43 | 0.81 | (0.53-1.23) |
| Q4 | 1,175 | 0.91 | (0.83-1.00) |  | 431 | **0.83** | **(0.72-0.96)** | 101 | 0.86 | (0.63-1.16) |  | 131 | 0.81 | (0.62-1.04) |  | 51 | 0.88 | (0.57-1.36) |
| *p for trend* |  | **0.042** |  |  |  | **0.029** |  |  | 0.447 |  |  |  | 0.076 |  |  |  | 0.564 |  |

Abbreviations: HR, hazard ratio; 95% CI, 95% confidence intervals; Q, quartile.

^1^ Cox proportional hazard models were used.

^2^ Adjusted for age (years, continuous); public health center area; smoking status (never, former, current), alcohol intake (no, >0-<150 g/w, 150-<300 g/w, 300+g/w), BMI (<25, 25 - <27, 27-<30, 30+), quartiles of metabolic equivalent task-hours/d, history of hypertension, history of diabetes, total energy intake, intakes of fruits, vegetables, fish, dairy products, egg, sodium and total fat (continuous).

^3^ Additionally adjusted for intake of chicken.

^4^ Additionally adjusted for intakes of pork, processed meat and chicken.

^5^ Additionally adjusted for intakes of beef, processed meat and chicken.

^6^ Additionally adjusted for intakes of beef, pork and chicken.

^7^ Additionally adjusted for intake of red meat.
