# Supplementary material for: Association between meat intake and mortality due to all-cause and major causes of death in a Japanese population
Source: PLoS One. 2020 Dec 15;15(12):e0244007. doi: 10.1371/journal.pone.0244007 (PMC7737902; doi:10.1371/journal.pone.0244007)
Supplement: S2 Table — (DOCX) [file pone.0244007.s003.docx]

**S2 Table. Adjusted hazard ratios of mortality by meat consumption status after excluding deaths within 5 years (women)**

|  | All-cause | | |  | Cancer | | |  | Cerebrovascular Disease | | |  | Heart Disease | | |  | Colorectal Cancer | | |
| --- | --- | --- | --- | --- | --- | --- | --- | --- | --- | --- | --- | --- | --- | --- | --- | --- | --- | --- | --- |
|  | Cases | HR^1,2^ | 95% CI |  | Cases | HR^1,2^ | 95% CI |  | Cases | HR^1,2^ | 95% CI |  | Cases | HR^1,2^ | 95% CI |  | Cases | HR^1,2^ | 95% CI |
| All meat |  |  |  |  |  |  |  |  |  |  |  |  |  |  |  |  |  |  |  |
| Q1 | 848 | 1.00 |  |  | 311 | 1.00 |  |  | 102 | 1.00 |  |  | 103 | 1.00 |  |  | 34 | 1.00 |  |
| Q2 | 712 | 0.98 | (0.88-1.09) |  | 272 | 0.98 | (0.83-1.17) |  | 63 | **0.67** | **(0.48-0.93)** |  | 90 | 1.01 | (0.75-1.37) |  | 32 | 1.01 | (0.61-1.68) |
| Q3 | 699 | 1.01 | (0.90-1.13) |  | 292 | 1.13 | (0.94-1.35) |  | 60 | **0.60** | **(0.42-0.87)** |  | 77 | 0.90 | (0.64-1.25) |  | 40 | 1.26 | (0.74-2.14) |
| Q4 | 748 | 1.07 | (0.92-1.23) |  | 261 | 1.06 | (0.83-1.34) |  | 61 | **0.52** | **(0.32-0.83)** |  | 99 | 1.04 | (0.70-1.54) |  | 36 | 1.16 | (0.59-2.29) |
| *p for trend* |  | 0.423 |  |  |  | 0.357 |  |  |  | **0.003** |  |  |  | 0.898 |  |  |  | 0.492 |  |
| Red meat^3^ |  |  |  |  |  |  |  |  |  |  |  |  |  |  |  |  |  |  |  |
| Q1 | 862 | 1.00 |  |  | 314 | 1.00 |  |  | 96 | 1.00 |  |  | 107 | 1.00 |  |  | 36 | 1.00 |  |
| Q2 | 694 | 0.95 | (0.85-1.06) |  | 274 | 1.00 | (0.84-1.19) |  | 73 | 0.87 | (0.62-1.20) |  | 81 | 0.86 | (0.63-1.17) |  | 33 | 0.97 | (0.58-1.60) |
| Q3 | 705 | 1.03 | (0.92-1.16) |  | 277 | 1.10 | (0.91-1.33) |  | 59 | 0.69 | (0.47-1.00) |  | 84 | 0.94 | (0.67-1.30) |  | 36 | 1.04 | (0.61-1.78) |
| Q4 | 746 | 1.05 | (0.90-1.21) |  | 271 | 1.13 | (0.89-1.44) |  | 58 | **0.59** | **(0.36-0.96)** |  | 97 | 0.95 | (0.63-1.43) |  | 37 | 0.98 | (0.49-1.95) |
| *p for trend* |  | 0.407 |  |  |  | 0.244 |  |  |  | **0.020** |  |  |  | 0.850 |  |  |  | 0.968 |  |
| Beef^4^ |  |  |  |  |  |  |  |  |  |  |  |  |  |  |  |  |  |  |  |
| Q1 | 923 | 1.00 |  |  | 324 | 1.00 |  |  | 96 | 1.00 |  |  | 124 | 1.00 |  |  | 37 | 1.00 |  |
| Q2 | 692 | 0.96 | (0.87-1.07) |  | 273 | 0.96 | (0.81-1.14) |  | 74 | 0.99 | (0.71-1.38) |  | 75 | 0.83 | (0.61-1.13) |  | 35 | 1.10 | (0.67-1.79) |
| Q3 | 682 | 0.98 | (0.88-1.09) |  | 277 | 1.04 | (0.87-1.24) |  | 62 | 0.87 | (0.61-1.24) |  | 82 | 0.97 | (0.72-1.31) |  | 40 | 1.17 | (0.71-1.92) |
| Q4 | 710 | 1.02 | (0.91-1.14) |  | 262 | 1.02 | (0.84-1.24) |  | 54 | 0.78 | (0.52-1.15) |  | 88 | 0.96 | (0.70-1.31) |  | 30 | 0.91 | (0.53-1.58) |
| *p for trend* |  | 0.823 |  |  |  | 0.675 |  |  |  | 0.174 |  |  |  | 0.930 |  |  |  | 0.876 |  |
| Pork^5^ |  |  |  |  |  |  |  |  |  |  |  |  |  |  |  |  |  |  |  |
| Q1 | 818 | 1.00 |  |  | 317 | 1.00 |  |  | 82 | 1.00 |  |  | 96 | 1.00 |  |  | 36 | 1.00 |  |
| Q2 | 692 | 0.96 | (0.86-1.07) |  | 269 | 0.94 | (0.79-1.12) |  | 69 | 0.88 | (0.62-1.25) |  | 89 | 1.00 | (0.74-1.37) |  | 35 | 1.07 | (0.65-1.76) |
| Q3 | 712 | 0.99 | (0.88-1.11) |  | 263 | 0.94 | (0.78-1.14) |  | 67 | 0.94 | (0.65-1.35) |  | 78 | 0.82 | (0.59-1.15) |  | 34 | 0.94 | (0.55-1.60) |
| Q4 | 785 | 0.96 | (0.84-1.10) |  | 287 | 1.01 | (0.81-1.26) |  | 68 | 0.74 | (0.47-1.16) |  | 106 | 0.88 | (0.61-1.28) |  | 37 | 0.88 | (0.47-1.64) |
| *p for trend* |  | 0.739 |  |  |  | 0.985 |  |  |  | 0.284 |  |  |  | 0.327 |  |  |  | 0.612 |  |
|  |  |  |  |  |  |  |  |  |  |  |  |  |  |  |  |  |  |  |  |
| Processed meat^6^ |  |  |  |  |  |  |  |  |  |  |  |  |  |  |  |  |  |  |  |
| Q1 | 967 | 1.00 |  |  | 344 | 1.00 |  |  | 92 | 1.00 |  |  | 121 | 1.00 |  |  | 45 | 1.00 |  |
| Q2 | 681 | 0.90 | (0.81-1.00) |  | 261 | 0.89 | (0.75-1.05) |  | 72 | 1.10 | (0.79-1.53) |  | 79 | 0.89 | (0.66-1.20) |  | 27 | 0.68 | (0.41-1.13) |
| Q3 | 666 | 0.97 | (0.87-1.08) |  | 250 | 0.94 | (0.79-1.12) |  | 68 | 1.15 | (0.81-1.64) |  | 86 | 1.09 | (0.81-1.47) |  | 23 | 0.65 | (0.38-1.12) |
| Q4 | 693 | 1.05 | (0.94-1.18) |  | 281 | 1.17 | (0.97-1.40) |  | 54 | 0.95 | (0.63-1.42) |  | 83 | 1.09 | (0.79-1.50) |  | 47 | 1.38 | (0.84-2.26) |
| *p for trend* |  | 0.372 |  |  |  | 0.130 |  |  |  | 0.991 |  |  |  | 0.442 |  |  |  | 0.251 |  |
| Chicken^7^ |  |  |  |  |  |  |  |  |  |  |  |  |  |  |  |  |  |  |  |
| Q1 | 831 | 1.00 |  |  | 318 | 1.00 |  |  | 74 | 1.00 |  |  | 99 | 1.00 |  |  | 36 | 1.00 |  |
| Q2 | 743 | 1.03 | (0.92-1.14) |  | 297 | 1.01 | (0.86-1.19) |  | 77 | 1.14 | (0.81-1.59) |  | 90 | 1.08 | (0.80-1.46) |  | 38 | 1.09 | (0.67-1.76) |
| Q3 | 689 | 0.96 | (0.86-1.07) |  | 276 | 0.96 | (0.81-1.14) |  | 68 | 1.01 | (0.71-1.44) |  | 78 | 0.99 | (0.73-1.36) |  | 31 | 0.96 | (0.57-1.61) |
| Q4 | 744 | 0.97 | (0.87-1.09) |  | 245 | 0.86 | (0.71-1.03) |  | 67 | 0.84 | (0.57-1.24) |  | 102 | 1.13 | (0.83-1.55) |  | 37 | 1.12 | (0.66-1.88) |
| *p for trend* |  | 0.395 |  |  |  | 0.103 |  |  |  | 0.339 |  |  |  | 0.546 |  |  |  | 0.799 |  |

Abbreviations: HR, hazard ratio; 95% CI, 95% confidence intervals; Q, quartile.

^1^ Cox proportional hazard models were used.

^2^ Adjusted for age (years, continuous); public health center area; smoking status (never, former, current), alcohol intake (no, >0-<150 g/w, 150-<300 g/w, 300+g/w), BMI (<25, 25 - <27, 27-<30, 30+), quartiles of metabolic equivalent task-hours/d, history of hypertension, history of diabetes, total energy intake, intakes of fruits, vegetables, fish, dairy products, egg, sodium and total fat (continuous).

^3^ Additionally adjusted for intake of chicken.

^4^ Additionally adjusted for intakes of pork, processed meat and chicken.

^5^ Additionally adjusted for intakes of beef, processed meat and chicken.

^6^ Additionally adjusted for intakes of beef, pork and chicken.

^7^ Additionally adjusted for intake of red meat.
