# Supplementary material for: Association between meat intake and mortality due to all-cause and major causes of death in a Japanese population
Source: PLoS One. 2020 Dec 15;15(12):e0244007. doi: 10.1371/journal.pone.0244007 (PMC7737902; doi:10.1371/journal.pone.0244007)
Supplement: S3 Table — (DOCX) [file pone.0244007.s004.docx]

**S3 Table. Adjusted hazard ratios of mortality from ischemic heart disease and intracerebral hemorrhage by meat consumption status**

|  | Men | | | | | | |  | Women | | | | | | |
| --- | --- | --- | --- | --- | --- | --- | --- | --- | --- | --- | --- | --- | --- | --- | --- |
|  | Ischemic Heart Disease | | |  | Intracerebral Haemorrhage | | |  | Ischemic Heart Disease | | |  | Intracerebral Haemorrhage | | |
|  | Cases | HR^1,2^ | 95% CI |  | Cases | HR^1,2^ | 95% CI |  | Cases | HR^1,2^ | 95% CI |  | Cases | HR^1,2^ | 95% CI |
| All meat |  |  |  |  |  |  |  |  |  |  |  |  |  |  |  |
| Q1 | 109 | 1.00 |  |  | 51 | 1.00 |  |  | 50 | 1.00 |  |  | 41 | 1.00 |  |
| Q2 | 79 | 0.80 | (0.59-1.10) |  | 39 | 0.96 | (0.61-1.51) |  | 43 | 0.94 | (0.61-1.46) |  | 19 | 0.53 | (0.29-0.98) |
| Q3 | 80 | 0.84 | (0.60-1.18) |  | 37 | 0.98 | (0.60-1.61) |  | 39 | 0.86 | (0.54-1.38) |  | 23 | 0.73 | (0.40-1.35) |
| Q4 | 99 | 1.10 | (0.72-1.69) |  | 54 | 1.43 | (0.78-2.65) |  | 50 | 0.94 | (0.53-1.65) |  | 28 | 0.87 | (0.40-1.86) |
| *p for trend* |  | 0.897 |  |  |  | 0.373 |  |  |  | 0.709 |  |  |  | 0.609 |  |
| Red meat^3^ |  |  |  |  |  |  |  |  |  |  |  |  |  |  |  |
| Q1 | 114 | 1.00 |  |  | 51 | 1.00 |  |  | 50 | 1.00 |  |  | 37 | 1.00 |  |
| Q2 | 76 | 0.75 | (0.55-1.03) |  | 36 | 0.85 | (0.53-1.35) |  | 41 | 0.89 | (0.57-1.40) |  | 27 | 1.03 | (0.59-1.79) |
| Q3 | 73 | 0.74 | (0.52-1.04) |  | 42 | 1.09 | (0.67-1.78) |  | 46 | 1.02 | (0.64-1.63) |  | 19 | 0.76 | (0.39-1.49) |
| Q4 | 104 | 1.05 | (0.68-1.60) |  | 52 | 1.38 | (0.74-2.59) |  | 45 | 0.80 | (0.44-1.44) |  | 28 | 1.12 | (0.50-2.48) |
| *p for trend* |  | 0.779 |  |  |  | 0.293 |  |  |  | 0.632 |  |  |  | 0.907 |  |
| Beef^4^ |  |  |  |  |  |  |  |  |  |  |  |  |  |  |  |
| Q1 | 124 | 1.00 |  |  | 47 | 1.00 |  |  | 60 | 1.00 |  |  | 40 | 1.00 |  |
| Q2 | 79 | 0.76 | (0.56-1.02) |  | 45 | 1.14 | (0.73-1.76) |  | 33 | 0.73 | (0.46-1.17) |  | 26 | 0.94 | (0.54-1.64) |
| Q3 | 69 | 0.67 | (0.49-0.93) |  | 49 | 1.33 | (0.85-2.08) |  | 46 | 1.12 | (0.73-1.72) |  | 22 | 0.89 | (0.50-1.59) |
| Q4 | 95 | 0.89 | (0.64-1.23) |  | 40 | 1.07 | (0.65-1.79) |  | 43 | 0.94 | (0.60-1.48) |  | 23 | 0.82 | (0.43-1.54) |
| *p for trend* |  | 0.280 |  |  |  | 0.574 |  |  |  | 0.856 |  |  |  | 0.522 |  |
| Pork^5^ |  |  |  |  |  |  |  |  |  |  |  |  |  |  |  |
| Q1 | 99 | 1.00 |  |  | 53 | 1.00 |  |  | 39 | 1.00 |  |  | 33 | 1.00 |  |
| Q2 | 82 | 0.98 | (0.71-1.34) |  | 42 | 0.93 | (0.60-1.44) |  | 50 | 1.45 | (0.92-2.27) |  | 24 | 0.86 | (0.47-1.54) |
| Q3 | 88 | 1.00 | (0.71-1.39) |  | 35 | 0.77 | (0.47-1.26) |  | 41 | 1.06 | (0.65-1.74) |  | 22 | 0.89 | (0.47-1.69) |
| Q4 | 98 | 1.02 | (0.69-1.51) |  | 51 | 1.01 | (0.58-1.75) |  | 52 | 1.03 | (0.60-1.79) |  | 32 | 1.21 | (0.59-2.47) |
| *p for trend* |  | 0.916 |  |  |  | 0.779 |  |  |  | 0.807 |  |  |  | 0.681 |  |
| Processed meat^6^ |  |  |  |  |  |  |  |  |  |  |  |  |  |  |  |
| Q1 | 116 | 1.00 |  |  | 57 | 1.00 |  |  | 61 | 1.00 |  |  | 37 | 1.00 |  |
| Q2 | 92 | 1.07 | (0.80-1.43) |  | 46 | 0.92 | (0.60-1.39) |  | 42 | 0.85 | (0.55-1.30) |  | 23 | 1.07 | (0.61-1.90) |
| Q3 | 80 | 0.88 | (0.64-1.21) |  | 43 | 0.88 | (0.56-1.36) |  | 35 | 0.76 | (0.48-1.21) |  | 26 | 1.30 | (0.73-2.33) |
| Q4 | 79 | 0.84 | (0.59-1.18) |  | 35 | 0.73 | (0.44-1.21) |  | 44 | 0.95 | (0.61-1.49) |  | 25 | 1.24 | (0.65-2.35) |
| *p for trend* |  | 0.219 |  |  |  | 0.237 |  |  |  | 0.680 |  |  |  | 0.408 |  |
| Chicken^7^ |  |  |  |  |  |  |  |  |  |  |  |  |  |  |  |
| Q1 | 104 | 1.00 |  |  | 47 | 1.00 |  |  | 54 | 1.00 |  |  | 35 | 1.00 |  |
| Q2 | 81 | 0.97 | (0.71-1.31) |  | 47 | 1.13 | (0.73-1.74) |  | 45 | 0.97 | (0.63-1.48) |  | 28 | 0.91 | (0.53-1.57) |
| Q3 | 83 | 0.97 | (0.71-1.33) |  | 50 | 1.26 | (0.81-1.95) |  | 31 | 0.70 | (0.44-1.12) |  | 21 | 0.74 | (0.41-1.33) |
| Q4 | 99 | 1.09 | (0.79-1.50) |  | 37 | 0.93 | (0.56-1.53) |  | 52 | 1.02 | (0.66-1.58) |  | 27 | 0.77 | (0.42-1.41) |
| *p for trend* |  | 0.643 |  |  |  | 0.941 |  |  |  | 0.768 |  |  |  | 0.307 |  |

Abbreviations: HR, hazard ratio; 95% CI, 95% confidence intervals; Q, quartile.

^1^ Cox proportional hazard models were used.

^2^ Adjusted for age (years, continuous); public health center area; smoking status (never, former, current), alcohol intake (no, >0-<150 g/w, 150-<300 g/w, 300+g/w), BMI (<25, 25 - <27, 27-<30, 30+), quartiles of metabolic equivalent task-hours/d, history of hypertension, history of diabetes, total energy intake, intakes of fruits, vegetables, fish, dairy products, egg, sodium and total fat (continuous).

^3^ Additionally adjusted for intake of chicken.

^4^ Additionally adjusted for intakes of pork, processed meat and chicken.

^5^ Additionally adjusted for intakes of beef, processed meat and chicken.

^6^ Additionally adjusted for intakes of beef, pork and chicken.

^7^ Additionally adjusted for intake of red meat.
